# Comparison of the neuromuscular effects of two infusion rates of rocuronium in anesthetized pigs

**DOI:** 10.1186/s13028-022-00658-7

**Published:** 2022-12-15

**Authors:** Kamilla Pedersen, Linda Loisa Kruhøffer, Jens Lykkesfeldt, Birgitte Saima Kousholt

**Affiliations:** 1 Section of Experimental Animal Models, Department of Veterinary and Animal Sciences, Faculty of Health and Medical Sciences, University of Copenhagen, Ridebanevej 9, 1870 Frederiksberg C, Denmark; 2 AUGUST Centre, Department of Clinical Medicine, Faculty of Health, Aarhus University, Palle Juul Jensens Blvd. 99 DK- 8200 Aarhus N and Nørrebrogade 44, Building 2B, DK-8000 Aarhus C, Denmark

**Keywords:** Acceleromyography, Neuromuscular blockade, Neuromuscular monitoring, Pharmacodynamics, Swine

## Abstract

**Background:**

Neuromuscular blocking agents are frequently administered to pigs used for research. In humans, administration of the drugs is not without risk and may result in accidental awareness under general anaesthesia and postoperative residual neuromuscular blockade that can lead to serious respiratory complications. Despite the extensive administration, the pharmacodynamics of neuromuscular blocking agents are not thoroughly studied in pigs. Therefore, this study investigates the neuromuscular response of two infusion rates of rocuronium, a commonly used non-depolarizing neuromuscular blocking agent. A group of 14 female Danish Landrace-Yorkshire-Duroc pigs used for supervised surgical training, weighing 40.3 ± 2.1 kg (mean ± SD), were included in the study. They received a loading dose of 0.85 mg/kg rocuronium intravenously followed by infusion of either 2.5 mg/kg/hour (L, low dose) or 5 mg/kg/hour (H, high dose) rocuronium for 30 min. Neuromuscular monitoring was performed with acceleromyography using train-of-four (TOF) stimulation. Onset time, time to reappearance of T1, T4, TOF ratio 90% and 100% were recorded.

**Results:**

All pigs in group H experienced loss of T1 throughout rocuronium infusion, whereas six out of seven pigs in group L had reappearance of T1 during rocuronium infusion, with additional reappearance of T4 in three of these pigs. The time to recovery of TOF ratio 90% was 14.0 ± 5.4 (L) and 21.7 ± 6.1 (H) minutes and recovery to TOF ratio 100% was 18.7 ± 6.5 (L) and 27.9 ± 9.2 min (H) (mean ± SD). Substantial inter-animal variation in neuromuscular recovery time was observed.

**Conclusion:**

The large inter-animal variation in pharmacodynamic profiles emphasizes that individual neuromuscular monitoring and titration to effect should be used routinely in research protocols that include rocuronium. In addition to other important measures, these actions are key in order to avoid overdosing and limit the risk of residual neuromuscular blockade.

**Supplementary Information:**

The online version contains supplementary material available at 10.1186/s13028-022-00658-7.

## Background

In biomedical research, neuromuscular blocking agents (NMBAs) are frequently administered during surgical procedures on pigs. Administration of NMBAs are reported in 20% of reviewed pig research studies from 2012 to 14 [1]. NMBAs cause muscle paralysis and their use complicates anaesthesia in numerous ways, including the monitoring of anaesthetic “depth”. Therefore, both neuromuscular transmission and anaesthetic depth should be monitored more diligently when these drugs are used. In humans, administration of NMBAs increases the risk of accidental awareness during general anaesthesia (AAGA), while correct administration and management decrease residual neuromuscular blockade (RNMB) in the postoperative phase [2, 3]. RNMB is a persisting partial neuromuscular blockade during and after recovery from general anaesthesia. It increases the risk of postoperative complications, such as hypoxia, weakness, decreased respiratory function and hence anaesthesia-related death in humans [4]. To our knowledge, the incidence of RNMB in experimental surgical pig studies from which the animals recover remains undisclosed, however, the incidence of RNMB in humans is estimated to be 40% [4]. The risk of RNMB correlates with NMBA dosing implying that overdosing must be avoided [5]. In humans, RNMB is defined as a train-of-four (TOF) ratio < 90% [6]. A recovery to ≥ 100% has, however, been suggested as the appropriate threshold when monitoring with acceleromyography [7]. Nevertheless, these reference values of RNMB apply to specific nerve-muscle units (NMUs) in human subjects and may not necessarily be associated with risk in non-human species when different NMUs are studied.

Dedicated monitoring equipment objectively assesses the degree of neuromuscular blockade and aid in correct administration. Different neuromuscular monitoring setups are available, and most often, it involves fixating a limb and avoiding any manipulation of the animal’s leg [8, 9]. Although monitoring is possible, NMBAs are rarely objectively monitored and often administered to clinically apparent effect in pigs [1]. The outcome could be overdosing and increased risk of RNMB [5]. In light of laboratory animal welfare and the 3Rs [10], more specifically refinement, initiatives to prevent RNMB are needed in recovery experiments. Apart from objective monitoring, such initiatives should include justified use of NMBAs [1] and the use of reversal agents when possible. Justified use of NMBAs and sufficient monitoring of anaesthesia depth are also of great importance in regards to AAGA, as the risk of AAGA when using NMBAs is a big concern as a refinement confounder.

Rocuronium is an aminosteroid and a non-depolarizing NMBA with a short onset time and intermediate duration of action [11]. These features render it popular for use in patients to facilitate endotracheal intubation. Rocuronium has species-specific and muscle-specific differences in sensitivity, e.g., pigs are less sensitive than rabbits, cats and humans [12–14] and the sensitivity in humans is also known to be affected by multiple factors including anaesthetic drugs, age, sex and body composition [15–18]. In spite of the extensive use of NMBAs in pigs, knowledge of the porcine-specific pharmacodynamics of rocuronium is still limited. Thus, the aim of this study was to investigate the neuromuscular effects of two infusion rates of rocuronium.

## Methods

### Animals

This study was conducted in accordance with Directive 2010/63/EU and approved by the Danish Animal Experiments Inspectorate (license no. 2016-15-0201-01029), and reporting follows the ARRIVE guidelines. Fourteen female crossbred pigs (Danish Landrace-Yorkshire-Duroc) used for supervised surgical training and weighing 40.3 ± 2.1 kg (mean ± SD) were included in the study. Pigs were acclimatized for one week and housed in a 12-hour light/dark cycle. Temperature was maintained between 18 and 24 ºC and animals were fed a commercial fiber-supplemented diet and had water *ad libitum* and access to hay, straw and environmental enrichment. Food, but not hay, straw and water, was withheld for 16 h prior to anaesthesia.

### Study design

The study was performed as a randomized and assessor-blinded study. Animals were randomly allocated to one of two groups, Low (L) or High (H) dose, by a computer-generated randomization table (Microsoft Excel 365; Microsoft Corporation, WA, USA). Assessor-blinding was achieved by help from a laboratory technician, who mixed the dose of rocuronium according to weight with an isotone 9 mg/mL saline infusion (Fresenius Kabi, Denmark) to a total of 25 mL and delivered the syringe to the blinded investigators at the operating theatre.

### Anaesthesia

To reduce the number of laboratory animals, we only included animals that were already anesthetized for surgical training purposes. The rocuronium infusion and neuromuscular measurements took place at the end of the training (after approximately 5–6 h of anaesthesia). Information on the surgical procedures can be found in Additional file 1. Anaesthesia was induced intramuscularly with a solution of 1.6 mg/kg Zoletil drymatter (Zoletil 50^®^Vet; Virbac, Denmark) dissolved in 1.6 mg/kg ketamine (Ketaminol Vet 100 mg/mL; Intervet, Denmark), 1.6 mg/kg xylazine (Rompun Vet 20 mg/mL; Bayer, Denmark) and 2.5 mg/kg butorphanol (Torbugesic Vet 10 mg/mL; Zoetis, Denmark). The pigs had intravenous auricular catheters in place, were intubated, placed in dorsal recumbency, and mechanically ventilated (Datex Ohmeda S5 Avance; GE Healthcare, IL, USA). Inspired/expired gases (O_2_, CO_2_ and sevoflurane) were monitored as part of the S5 Avance workstation. Inspired oxygen fraction was 40% and the positive end-expiratory pressure 5 cm H_2_O. Tidal volume was 10–15 mL/kg and respiratory rate 8–18 breaths/minute to maintain end-tidal CO_2_ between 4.5 and 5.6 kPa. Animals were kept anaesthetised with sevoflurane (2.4–2.8% end-tidal concentration) and a fentanyl (Fentanyl B. Braun 50 µg/mL; B. Braun, Denmark) infusion (15–20 µg/kg/hour). Additionally, eight out of 14 pigs were supplemented with 10–20 mg/kg ketamine (Ketaminol Vet 100 mg/mL; Intervet, Denmark) during the surgical training. Monitoring values were recorded manually every 15 min. An isotone 9 mg/mL saline infusion 10 mL/kg/hour, a urinary catheter (Rüsch Brilliant AquaFlate Glycerin Silicone size ch. 12; Teleflex, Pennsylvania, USA) and a rectal thermometer were already in place. An OxyTip (Datex Ohmeda; GE Healthcare, IL, USA) was repositioned on either tongue or tail to measure pulse rate and oxygen saturation (SpO_2_).

Blood-gas analyses (ABL 900; Radiometer, Denmark) were performed before the neuromuscular measurements and at the end of the study. If core temperature < 38 °C, a heating blanket (Warm Touch; Nellcor, Minneapolis, USA) was applied. A skin thermometer (2000T; Digitron, England) was placed near the neuromuscular monitoring electrodes. Blood pressure was measured with a 12–19 cm cuff (Critikon Dura-Cuf; GE Healthcare, IL, USA) placed either on the carpus of the left thoracic limb or just distal to the hock on the left pelvic limb. A supplemental bolus of 6.5 µg/kg fentanyl was administered 5–10 min prior to starting neuromuscular stimulation to ensure sufficient analgesia.

### Neuromuscular monitoring

**Fig. 1:**
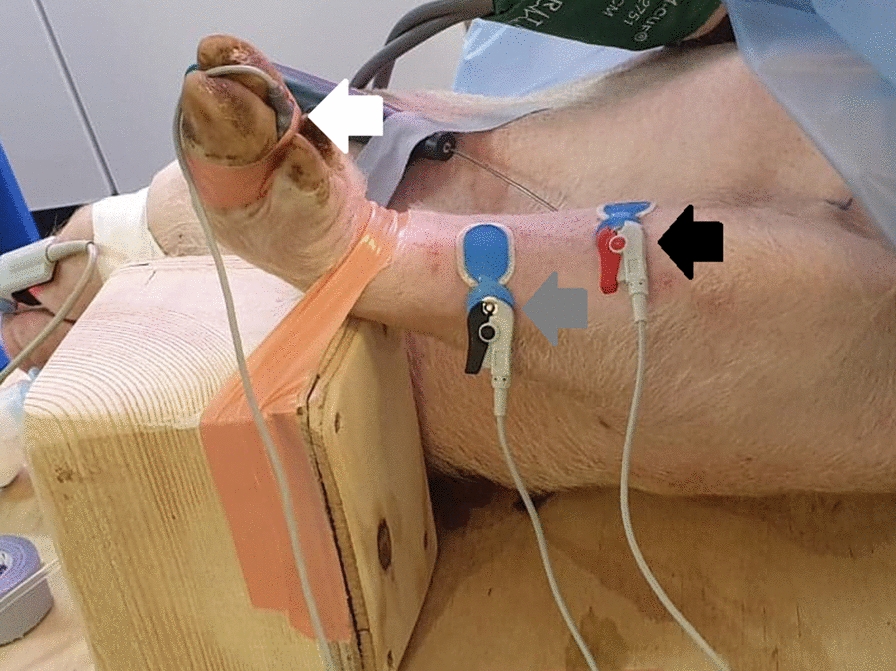
Experimental setup. The leg was fixed to the box with tape across the palmar side of the carpus. Electrodes were placed on the caudal side of the antebrachium. The transducer was fixed between the third and fourth digit. Grey arrow: Cathode. Black arrow: Anode. White arrow: Transducer

An acceleromyograph (Stimpod NMS450X; Xavant Technology, South Africa) was connected to a computer with a software for collecting neuromuscular data consisting of TOF ratios and accelerometer magnitudes (Intelli Cable Software: NMShow; Xavant Technology, South Africa). Neuromuscular monitoring was performed according to international guidelines for good clinical research practice in pharmacodynamic studies of NMBAs [19] except for calibration of twitch height, which is not necessary with the Stimpod NMS450X. Fixation and electrode placement were modified from Madsen et al. [8] and refined during pilot studies to ensure a stable TOF ratio baseline. The skin was cleansed, shaved, and rubbed with 85% ethanol. The acceleration transducer was fixed between the third and fourth digit. The ulnar nerve was located using the nerve mapping pen (Stimpod NMS450X; Xavant Technology, South Africa). Pediatric electrodes (Ambu^®^ BlueSensor N; Ambu, England) were used. The cathode placed over the ulnar nerve 10–12 cm distally of the elbow joint, and the anode was placed 4–6 cm proxomedially from here, measured from center to center (Fig. 1).

Four TOF stimulations of 50 mA were applied, followed by 50 Hz tetanic stimulation for five seconds. Supramaximal stimulation mode on the Stimpod NMS450X was applied to determine the individual current needed for supramaximal stimulation. The supramaximal stimulation mode consisted of 16 1 Hz square wave pulses of 200 µs and increasing intensities (5–80 mA), and the device determined the required magnitude of the current. This current was transferred to TOF mode stimulating with a 2 Hz frequency with a 15 s refractory period. The stability of the TOF ratio baseline was manually assessed by evaluating whether the TOF ratio values over a period of 5 min were homogenous. If the TOF ratios varied too much, baseline monitoring was prolonged until stability for 5 min was obtained. The coefficient of variation during baseline monitoring was calculated post hoc to ensure that our initial assessment of baseline stability was correct. When the TOF ratio baseline was stable and absence of pedal, septal and palpebral reflexes ensured, rocuronium (Rocuronium Hameln 10 mg/mL; Hameln Pharma, England) was administered.

Neuromuscular blockade was induced with a loading dose of 0.85 mg/kg rocuronium that was administered within five seconds. Neuromuscular blockade was maintained with infusion of either 2.5 mg/kg/hour (L) or 5.0 mg/kg/hour (H) rocuronium for 30 min. As the elimination half-life of rocuronium in pigs has not been established, we based the dosing regimens on a pilot infusion study, experience gained from a previous experiment [20], and clinical literature in humans [21, 22]. Collectively, we expected the infusion time of 30 min to be sufficient to approximate steady state and enable the comparison of the two infusion doses used. Hereafter, spontaneous recovery of neuromuscular function was allowed, and times to reappearance of T1, T4, TOF ratio 90% and 100% were recorded (Table 1). TOF terminology is used in accordance with current guidelines [19]. Briefly, T1 is the first twitch in the train-of-four and the last twitch to disappear and the first to reappear when using non-depolarizing NMBAs. T4 is the fourth and last twitch in the train-of-four and is the first twitch to disappear and the last to reappear [23]. Neuromuscular monitoring continued until three consecutive measurements of TOF ratio ≥ 100% were obtained. After completion of monitoring, absence of pedal, septal and palpebral reflexes was checked again, and the pigs were euthanized with 75 mg/kg pentobarbital (Exagon vet 400 mg/mL; Salfarm, Denmark) intravenously.

**Table 1:** Definitions of neuromuscular monitoring variables

Terminology	Definition
Onset time	Time from injection of NMBA until complete disappearance of all twitches.
Reappearance of T1	Time from termination of NMBA infusion until reappearance of the first twitch (T1) in the TOF.
Reappearance of T4	Time from termination of NMBA infusion until reappearance of the fourth twitch (T4) in the TOF.
TOF ratio 90%	The neuromuscular recovery endpoint. Time from termination of NMBA infusion until a TOF ratio of 90%.
TOF ratio 100%	The potential neuromuscular recovery endpoint when using acceleromyography. Time from termination of NMBA infusion until a TOF ratio of 100%.

### Statistical analysis

Statistical analyses were performed with GraphPad Prism 8.3.0 (GraphPad Software, California, USA). Onset and recovery times were rounded off to nearest quarter of a minute. TOF ratios are actual ratios and have not been normalised [24]. QQ-plots confirmed normal distribution and the F-test supported the assumption of variance homogeneity for time to recovery of TOF ratio 90% and 100%. These data were analysed with the one-tailed Student’s t-test. The Holm multiple comparisons test was applied for post-hoc testing. Monitoring values i.e. pulse rate, respiratory rate, etc. were likewise checked for normal distribution and variance homogeneity. Initial monitoring values were recorded just before rocuronium dosing and analysed with a two-tailed Student’s t-test. General monitoring data were pooled from all animals across the entire monitoring period, and analysed with a repeated measures mixed-effects analysis due to missing values. Alpha was set to 0.05. Recovery to TOF ratio 90% and 100% are presented as mean ± SD, while the other neuromuscular data and monitoring values are presented as medians (minimum-maximum).

### Sample size

We used data from a pilot infusion study and previously published data on rocuronium in pigs to make an estimate of effect size and standard deviation [12, 13, 25–28]. A power of 80% and a confidence interval of 95% led to a sample size estimate of minimum six pigs per group.

### Exclusion criteria

Pigs were not included in the study if the performed surgical procedure completely compromised elimination of rocuronium, e.g. liver excision or bilateral nephrectomy. Additionally, pigs were excluded if they were too unstable to continue anaesthesia, e.g., decreasing blood pressure, elevated pulse rate or quickly increasing core temperature indicating sepsis etc.

## Results

All pigs were included in the study leading to a total of seven pigs per group. Physiological variables from baseline and during the entire monitoring period are presented in Table 2. Systolic arterial pressure was lower in group L than in group H at both the beginning and throughout the experiment. Skin temperature was significantly higher in group L than in group H at the beginning of the experiment but not throughout the experiment, while core temperature was significantly higher in group L than in group H at both the beginning and throughout the experiment. Pulse rate, respiration rate, SpO_2_, end-tidal CO_2_ and pH were not significantly different between the groups. Individual monitoring data as well as details on the surgical procedures each pig underwent can be found in Additional file 1.

**Table 2:** Monitoring values

Group	PR (beats/min)	RR (breaths/min)	SpO_2_ (%)	EtCO_2_ (kPa)	pH	SAP (mmHg)	T, skin (ºC)	T, central (ºC)
L, initial	85 (62–103)	14 (12–16)	100 (98–100)	5.1 (4.7–5.6)	7.47 (7.42–7.58)	66 (53–78)*	36.5 (36.0–38.0)**	37.7 (36.6–38.4)**
H, initial	79 (71–81)	12 (11–13)	100 (100–100)	5.2 (5.0-5.4)	7.45 (7.25–7.53)	82 (77–90)	34.5 (34.5–35.7)	36.3 (35.0-36.7)
L, general	87 (62–124)	14 (12–16)	100 (96–100)	5.1 (4.6–5.9)	7.45 (7.38–7.58)	69 (53–86)^#^	36.0 (35.1–38.0)	38.0 (36.6–38.4)^##^
H, general	75 (64–101)	12 (10–17)	100 (99–100)	5.0 (4.6–5.8)	7.43 (7.18–7.53)	83 (61–97)	35.6 (33.4–37.3)	36.5 (34.5–37.7)

Values displayed as median (minmum-maximum). pH is temperature corrected. Initial monitoring values were recorded just before rocuronium dosing. General monitoring values were recorded every 15 min throughout the experiment and pooled for all animals. n = 7 per group. For individual monitoring data please refer to Additional file 1

*PR* pulse rate. *RR* respiration rate, *SpO*_*2*_ oxygen saturation. *EtCO*_*2*_ end-tidal carbon dioxide, *SAP* systolic arterial pressure, *T skin* skin temperature, *T central* central temperature

^*^P < 0.05 when compared to H, initial

^**^P < 0.01 when compared to H, initial

^#^P < 0.05 when compared to H, general

^##^P < 0.01 when compared to H, general

**Fig. 2:**
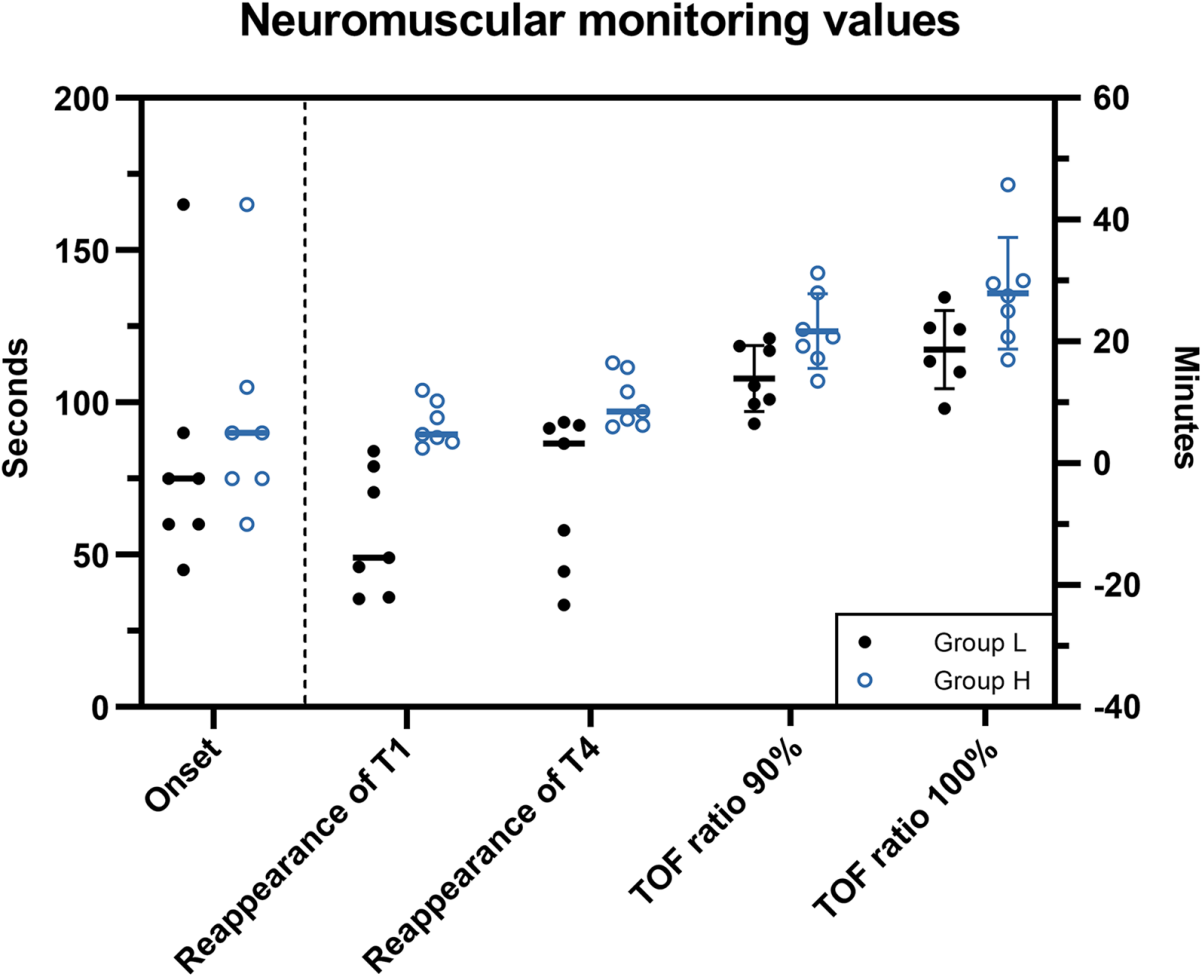
Neuromuscular monitoring values. Onset time is determined by the left y-axis, while the remaining values are determined by the right y-axis (indicated by the dashed line). Onset time, reappearance of T1 and T4 values are displayed as individual values with the median. TOF ratio 90% and 100% are displayed as individual values with the mean ± SD. Negative recovery time values indicate that recovery happened before termination of infusion. For definitions of the neuromuscular monitoring variables please refer to Table 1

In this experimental setup, the median current needed for supramaximal stimulation was 65 mA with a range of 45–80 mA. The TOF ratio baseline was 108 ± 5.2% for all pigs. The TOF ratio baseline was stable for 5 min before administration of rocuronium and had a median coefficient of variation of 2.9% with a range of 1.6–5.6% for all pigs. In group H, T1 disappeared in all animals subsequent to administration of the loading dose, and loss of T1 was maintained throughout the infusion period. In group L, T1 disappeared in four out of seven pigs after administration of the loading dose, but complete neuromuscular blockade was not present in the remaining three pigs. However, lack of complete blockade was interpreted to be due to direct muscle stimulation as opposed to an inefficient loading dose as described below. The infusion rate in group L was not sufficient to maintain loss of T1 nor loss of T4 during rocuronium infusion in all of the pigs. In detail, six pigs had reappearance of T1 and three of these animals additionally experienced reappearance of T4 during rocuronium infusion (indicated with negative neuromuscular recovery time values, Fig. 2). One pig was euthanized before TOF ratio 100% could be reached (#4, Group L), because of a sudden hemodynamic instability towards the end of the study. Due to a technical error one pig (#2, Group H) had rocuronium infusion for 32 min.

Pigs in group H were significantly slower to recover to a TOF ratio 90% (21.7 ± 6.1 vs. 14.0 ± 5.4 min, P = 0.03) and 100% (27.9 ± 9.2 vs. 18.7 ± 6.5 min, P = 0.03) after termination of infusion than group L. Both groups displayed inter-animal variations in all neuromuscular recovery variables and onset time (Fig. 2).

Three pigs (#1, 4 and 8, Group L) did not display complete neuromuscular blockade at any point after administration of the loading dose or during infusion. The accelerometer magnitude was small and almost constant subsequent to loading dose administration and during part of the infusion time. Most importantly, no TOF fade was present during this period with T4 exceeding or being equal to T1. These characteristics were interpreted to be caused by direct muscle stimulation as described in the guidelines for human pharmacodynamic studies of NMBAs [19]. Therefore, the three datasets were adjusted by subtracting the small accelerometer magnitude present during blockade from all measured twitches, as recommended [19]. This adjustment did not influence TOF ratios, and thus the datasets were regarded as valid. After post-hoc adjustment, T1 was considered absent when T4 equalled or exceeded T1 subsequent to loading dose administration and reappeared when T1 exceeded T4, enabling calculation of onset time and recovery variables in the same way as the remaining datasets.

## Discussion

The aim of our study was to investigate the neuromuscular response to two relevant rocuronium infusion doses. As expected, pigs receiving 5.0 mg/kg/hour rocuronium for 30 min were slower to recover from neuromuscular blockade than pigs receiving 2.5 mg/kg/hour. More importantly, however, both groups showed large inter-animal variation in neuromuscular recovery times. This finding is in congruence with the overall findings in previous studies in humans [21, 22], pigs [25, 26] and horses [29]. The present study was controlled for sex, age, and body composition, variables known to influence rocuronium pharmacodynamics [15–17]. As expected during surgery, variations in core temperature, blood pH, and pharmaceuticals (here supplementation with ketamine) were present, and may have further increased variability in neuromuscular recovery times. Low core temperature and in particular hypothermia increases the duration of action of NMBAs [30, 31], and since the median core temperatures of group H were lower than group L this may have further increased neuromuscular recovery times in group H. Eight pigs had posthypercapnic alkalemia caused by the preceding laparoscopy [32] and alkalemia decreases the sensitivity to rocuronium in in vitro experiments conducted on rat tissue [33]. Thus, differences in blood pH may have increased variability in neuromuscular recovery time. Nevertheless, neither core temperature nor pH correlated significantly with neuromuscular recovery time. Another factor possibly affecting the neuromuscular recovery time was the administration of ketamine to eight out of 14 pigs during surgery but before this experiment was initiated. Ketamine has been reported to potentiate rocuronium in cats [34], and even though the clinical duration of ketamine is short in pigs [35], the elimination half time is about two hours [36]. To disclose whether ketamine supplementation introduces significant variation in the neuromuscular recovery time, such interactions should be further investigated in the relevant species. Moreover, we investigated the neuromuscular recovery of the thoracic limb measured by acceleromyography according to Madsen et al. [8]. However, when investigating neuromuscular recovery in future studies, it is also advisable to take into account that different NMUs have different sensitivity to NMBAs and are accordingly more or less relevant for RNMB.

Based on a pilot infusion study and a previous study protocol using rocuronium infusion [20], it was anticipated that 2.5 mg/kg/hour rocuronium would be sufficient to maintain loss of T1. However, this dose was inadequate in the majority of the pigs in the present study. The age and body weight of the animals may explain the difference in sensitivity [15]. The pilot pig and the pigs in Tjørnild et al.’s study [20] were the same breed but were older and weighed 80 kg. Thus, the dose of 2.5 mg/kg/hour may be suitable for older and larger pigs, while a higher dose will most likely be necessary to maintain loss of T1 in younger and smaller animals. Moreover, as we did not measure rocuronium concentrations in the present study, we have not been able to confirm that 30 min of infusion is in fact sufficient to approximate steady state. To disclose this, further studies are needed. Based on the results of this study, an initial dose of 2.5 mg/kg/hour and titration to effect is recommended. Titration should be done with aid from neuromuscular monitoring to objectively assess the degree of neuromuscular blockade and thereby avoid overdosing [5]. This recommendation is supported by studies in humans, where individual neuromuscular monitoring significantly decreases the risk of RNMB [2]. Furthermore, monitoring in general supports increased awareness to minimize possible complications pre- and postoperatively [37].

Because of the study design, the findings in this study may primarily be representative of neuromuscular recovery after an extensive surgical intervention. Rocuronium was administered later than usual in a clinical or experimental setting, and since the pigs underwent surgical procedures in advance, pharmacokinetics and –dynamics may have been altered. Furthermore, the different surgical interventions add to the heterogeneity of the study material, and may have influenced the neuromuscular recovery times. Nevertheless, this study may be able to increase awareness of the potentially large differences in individual neuromuscular recovery times. Another possible limitation is the high incidence of direct muscle stimulation. Since direct muscle stimulation can be corrected for, it does not directly influence the TOF ratios, but it does generate an extra level of post-hoc data manipulation and may affect evaluation of the absence or presence of twitches. It was not possible to identify why these exact animals had direct muscle stimulation, and changing or moving the electrodes did not solve the problem. Perhaps the problem is related to the monitoring device, choice of nerve to be stimulated, electrodes or rocuronium dose. Nevertheless, the setup was very similar to the acceleromyography setup in Madsen et al. [8], where no direct muscle stimulation was reported. In future neuromuscular monitoring of pigs, a setup similar to one used in dogs could be investigated [38]. Here, the ulnar nerve is stimulated with needle electrodes placed over the medial epicondyle of the humerus, thus not passing the electrical current directly over the muscle. A similar setup may prevent direct muscle stimulation from occurring.

The significance of the present study is that the sensitivity to rocuronium varies substantially between pigs. This considerable variation stresses the point that neuromuscular monitoring and titration to effect need to be routine when rocuronium is part of the research protocol. However, routine implementation of neuromuscular monitoring is not without its challenges. Currently available monitoring systems, whether they are based on mechanomyography, electromyography or acceleromyography [8, 9, 27], are more cumbersome to mount in pigs than in humans. Moreover, the reference values of RNMB apply to specific NMUs in humans and further investigations on how these correspond to non-human species are needed. The absence of neuromuscular monitoring in pigs may also be due to lack of focus on its importance [1], and other veterinary fields face similar challenges [39]. In addition to better monitoring, implementation of reversal drugs e.g. sugammadex or neostigmine may significantly lower the risk of RNMB and improve the postoperative phase, but the pharmacodynamics in pigs still need further investigation. Lastly, tighter regulation of the use of NMBAs, better justification when applied, and greater awareness of the serious complications that may arise, are important measures to take when aiming to reduce the risk of RNMB in experimental pig studies.

## Conclusion

The low dose rocuronium infusion of 2.5 mg/kg/hour was insufficient to maintain loss of T1 in six out of seven pigs in group L. All pigs in group H receiving 5.0 mg/kg/hour experienced loss of T1 throughout rocuronium infusion. Considerable inter-animal variation in neuromuscular recovery time was present, underlining that neuromuscular monitoring should routinely be implemented to ensure correct dosing and limit the risk of residual neuromuscular blockade.

## Supplementary Information

**Additional file 1.** Individual monitoring values. Monitoring values from all pigs presented as median [minimum-maximum]. pH is temperature corrected and reported as start of protocol (S) and end of protocol (E). N/A: not available. PR: pulse rate. RR: respiration rate. SpO_2_: oxygen saturation. EtCO_2_: end-tidal carbon dioxide. SAP: systolic arterial pressure. T, skin: skin temperature. T, central: central temperature. Type of surgery: laparotomy (LT), laparoscopy (LS). Operations: urology with unilateral nephrectomy (Uro w/N), urology without nephrectomy (Uro w/o N), intestinal anastomosis (IA), hysterectomy (H), cholecystectomy (CC).

## Data Availability

The datasets used and/or analysed during the current study are available from the corresponding author on reasonable request.
