# Supplementary material for: Comparison of the neuromuscular effects of two infusion rates of rocuronium in anesthetized pigs
Source: Acta Vet Scand. 2022 Dec 15;64:38. doi: 10.1186/s13028-022-00658-7 (PMC9753331; doi:10.1186/s13028-022-00658-7)
Supplement: Supplementary file 1 — Additional file 1. Individual monitoring values. Monitoring values from all pigs presented as median [minimum-maximum]. pH is temperature corrected and reported as start of protocol (S) and end of protocol (E). N/A: not available. PR: pulse rate. RR: respiration rate. SpO2: oxygen saturation. EtCO2: end-tidal carbon dioxide. SAP: systolic arterial pressure. T, skin: skin temperature. T, central: central temperature. Type of surgery: laparotomy (LT), laparoscopy (LS). Operations: urology with unilateral nephrectomy (Uro w/N), urology without nephrectomy (Uro w/o N), intestinal anastomosis (IA), hysterectomy (H), cholecystectomy (CC). [file 13028_2022_658_MOESM1_ESM.pdf]

Additional file 1 Individual monitoring values

Pig number and group	PR (beats/min)	RR (breaths/min)	SpO_2 (%)	$EtCO_2$ (kPa)	pH	SAP (mmHg)	T, skin (^oC)	T, central (^oC)	Type of surgery	Major intervention
1, Group L	90 [87- 91]	16 [15- 16]	100	5.0 [4.8- 5.6]	S:7.415 E:7.488	76 [69-78]	37.1 [37.0- 37.2]	38.2 [37.9- 38.4]	LT	Uro w/N
2, Group H	85 [69- 101]	13 [12- 13]	100	4.9 [4.6- 5.6]	S:7.250 E:7.183	68 [64-76]	35.3 [34.3- 35.3]	36.6 [36.3- 36.8]	LT	Uro w/N
3, Group L	83 [80-85]	14 [13-14]	100 [99-100]	4.8 [4.6-5.2]	S:7.469 E:7.428	65 [56-72]	36.0 [35.1-36.6]	38.3 [38.0-38.4]	LS	Uro w/o N
4, Group L	108 [103-124]	14 [12-14]	100	5.1 [5.1-5.6]	S:7.445 E: N/A	75 [66-86]	35.7 [35.1-36.0]	37.1 [36.6-37.2]	LS	Unknown
5, Group H	82 [82-87]	11 [10-13]	100	5.0 [4.8-5.7]	S:7.447 E:7.404	83 [78-89]	35.0 [34.5-34.1]	35.4 [34.9-35.9]	LS	Uro w/N + CC
6, Group H	73 [64-77]	14 [11-14]	100	5.0 [4.9-5.8]	S:7.405 E:7.389	89 [82-94]	36.9 [34.5-37.3]	37.2 [36.5-37.6]	LS	Uro w/o N
7, Group H	74 [69-80]	11	100	4.9 [4.8-5.0]	S:7.466 E:7.457	94 [88-97]	35.5 [35.1-35.8]	35.6 [35.1-36.0]	LS	Uro w/N + CC
8, Group L	63 [62-65]	13	99 [99-100]	5.3 [5.1-5.9]	S:7.453 E:7.400	74 [65-77]	36.0 [35.4-36.3]	37.2 [36.7-37.6]	LS	IA + CC
9, Group H	73 [69-74]	15 [14-15]	100	5.0 [4.9-5.5]	S:7.446 E:7.420	83 [73-86]	36.5 [36.3-36.7]	37.3 [36.9-37.7]	LS	H + CC
10, Group L	87 [85-92]	15 [13-15]	100	4.9 [4.6-5.2]	S:7.469 E:7.423	69 [53-76]	37.0 [36.7-37.7]	38.0 [37.6-38.0]	LS	Unknown
11, Group L	81 [77-83]	13 [12-13]	98 [96-98]	5.4 [5.2-5.5]	S:7.511 E:7.479	66 [65-69]	35.9 [35.7-36.5]	37.8 [37.7-37.8]	LS	Uro w/o N
12, Group L	89 [89-103]	14 [14-15]	100	4.9 [4.8-5.3]	S:7.584 E:7.383	71 [62-73]	35.5 [35.1-37.9]	38.4 [38.3-38.4]	LS	Unknown
13, Group H	70 [68-70]	11 [10-11]	99	5.3 [5.2-5.5]	S:7.440 E:7.390	63 [61-70]	33.6 [33.4-34.0]	35.4 [34.5-36.2]	LS	Uro w/o N
14, Group H	78 [75-80]	16 [16-17]	99 [97-99]	4.8 [4.7-5.1]	S:7.525 E:7.508	86 [80-96]	36.0 [35.6-36.2]	37.5 [37.4-37.6]	LS	Uro w/N

Monitoring values from all pigs presented as median [minimum-maximum]. pH is temperature corrected and reported as start of protocol (S) and end of protocol (E). N/A: not available. PR: pulse rate. RR: respiration rate. SpO_2: oxygen saturation. $EtCO_2$: end-tidal carbon dioxide. SAP: systolic arterial pressure. T, skin: skin temperature. T, central: central temperature. Type of surgery: laparotomy (LT), laparoscopy (LS). Operations: urology with unilateral nephrectomy (Uro w/N), urology without nephrectomy (Uro w/o N), intestinal anastomosis (IA), hysterectomy (H), cholecystectomy (CC).
